# Brain-derived Neurotrophic Factor and Its Applications through Nanosystem Delivery

**DOI:** 10.22037/ijpr.2021.115705.15484

**Published:** 2021

**Authors:** Mengyao Xia, Tingting Zhao, Xiaolong Wang, Yang Li, Yanling Li, Tingting Zheng, Jiaxin Li, Yu Feng, Yongli Wei, Peng Sun

**Affiliations:** a *Department of Pharmacology, School of Pharmacy, Shandong University of Traditional Chinese Medicine, Ji’nan 250355, China.*; b *Center for Foreign Language Translation, College of Foreign Languages, Shandong University of Traditional Chinese Medicine, Ji’nan250355, China. *; c *Innovation Research Institute of Chinese Medicine, Shandong University of Traditional Chinese Medicine, Ji’nan 250355, China. *; d *Department of Drug Design, College of Intelligence and Information Engineering, Shandong University of Traditional Chinese Medicine, Ji’nan 250355, China. *; e *Affiliated Hospital of Shandong University of Traditional Chinese Medicine, Ji’nan 250014, China.*

**Keywords:** Brain-derived neurotrophic factor, Pathogenesis, Nanomaterials, Hydrogel, Drug delivery, Neuropsychiatric diseases

## Abstract

Brain-derived neurotrophic factor (*BDNF*) is a protein that performs a neurotrophic function. *BDNF* and its receptors are widely expressed in the nervous system and can promote the growth of neurons and the formation of neuronal synapses in the brain. Studies have shown that a lack of *BDNF* can lead to impairment of memory and cognitive functions, indicating that *BDNF* plays an important role in mental illness and neurodegenerative diseases. The combination of stem cells and *BDNF*-releasing nanomaterials holds great promise in regenerative medicine, especially in the treatment of neurological diseases. For example, Alzheimer’s disease, depression, Parkinson’s disease, spinal cord injury, etc. The combination of stem cell/pharmacologically active carrier and *BDNF*-nano/hydrogel provided a useful new type of local delivery tool for the treatment of the nervous system and other diseases. It can not only provide *BDNF* but also stem cells. These studies will provide a scientific basis for the development and application of *BDNF* in the future.

## Introduction

**Figure 1:**
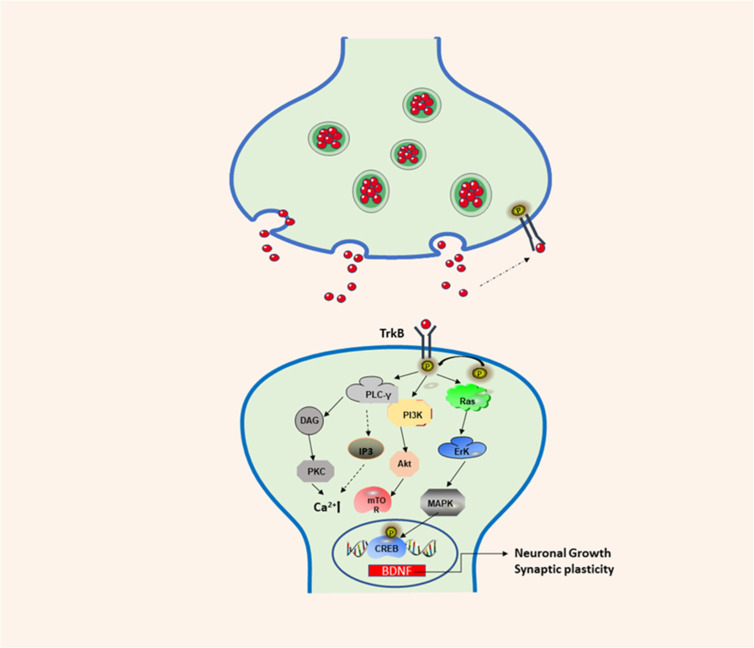
*BDNF*/*TrkB *signaling pathway involved in neuronal differentiation and synaptic plasticity. *TrkB*: Tropomyosin-receptor-kinaseB; *PI3K*: phos-phatidylinositol 3-kinase; *Akt*: protein kinase B; *Erk*: extracellular-signaling-regulated kinase; MAPK: mitogen-activated protein kinase; *CREB*:* cAMP* response element-binding protein; *PLC-γ*: phospholipase C-gamma; *DAG*: 1:2-diacylglycerol; *PKC*: protein kinase C

*BDNF* is a neurotrophic factor is a protein that supports the function of the central nervous system. *BDNF* is mainly distributed in the central nervous system, and is highly expressed in the hippocampus and frontal cortex (1, 2). In addition, it is also distributed in other areas, such as the peripheral nervous system, endocrine system, and bone and cartilage tissue (3-5). During the development of the nervous system, *BDNF* performs important functions by influencing cell differentiation, neuron development, growth and survival, neurogenesis, synapse formation and synaptic plasticity (5, 6). *BDNF* can bind to tyrosine kinase B (*TrkB*) to activate intracellular domains, promote *TrkB* autophosphorylation, and finally activate the three main pathways: *Ras-MAPK*, *PI3K*, and *PLC-γ *resulting in the activation of the* cAMP* response element-binding protein (*CREB*) (7-9) (Figure 1). *CREB *promotes the survival of nerve cells and improves synaptic plasticity and neurogenesis by increasing the expression of the *BDNF* gene and *BCL-2 *anti-apoptotic protein gene (10). *NF-κB *(nuclearfactor-kappa B) is one of the major factors of inflammatory activation, and it is a transcriptional factor, which can induce the expression of pro-apoptotic and anti-apoptotic genes and *BDNF*.In addition, mature *BDNF* (mature-*BDNF*, m-*BDNF*) and the *BDNF* precursor (pro-*BDNF*) can bind to the low-affinity p75 neurotrophin receptor (*p75NTR*) to produce corresponding biological activity (11). Recently, an increasing amount of research has been conducted on *BDNF*, and its genetic polymorphism has become a subject that is of great interest to researchers. The *BDNF* gene is located on chromosome 11p13-14. If the 66^th^ codon, guanine, in the coding region of the gene is replaced by adenine, methionine replaced valine, resulting in *BDNF* Val66Met polymorphism, which is closely associated with cognitive dysfunction (12). Studies have shown that a lack of *BDNF* can lead to mental disorders and neurodegenerative diseases (13). At present, stem cells are used to produce *BDNF* and for the targeted delivery of *BDNF* to treat certain diseases, but the main challenge is that drugs or stem cells cannot cross the blood-brain barrier (*BBB*). The use of appropriate nanomaterials for the targeted delivery of drugs or stem cells in which *BDNF* can be delivered to the lesion to release and maintain its biological activity has become an important scientific issue. Therefore, the identification of suitable carriers that can successfully target the delivery of *BDNF* will be necessary to develop novel strategies and a theoretical basis for the treatment of various brain diseases.

This paper reviewed the application of nano-drug delivery system loaded *BDNF* in the treatment of various diseases, which provided a new scientific basis for the development and application of *BDNF* carriers in the future.

The combined use of *BDNF* and *TrkB* enhances the autophosphorylation of *TrkB* and activates three different signaling pathways. The first is the *PI3K*/Akt-related pathway. *PI3K* induces the transcription of the *BDNF* gene by activating *BDNF* mRNA, and exerts anti-apoptotic and survival activities. Then, the *PI3K*/*Akt*/*mTOR *cascade regulates protein synthesis and cytoskeleton development, enhancing dendritic growth and branching. The second pathway is the *MAPK/Ras *pathway, which regulates the signal transduction pathway of protein synthesis and activates *Erk/CREB* related pathways. The third is the *PLC-γ*-dependent pathway, which activates calmodulin kinase, and *PKC*, increasing levels of *DAG *expression and calcium ion concentration, which in turn increases synaptic plasticity.

**The role of** ***BDNF*** **in diseases**

*The role of BDNF in mental illness and neurodegenerative diseases*

*Major depressive disorder*

Major depressive disorder is a common mental illness. At present, the mechanism by which combinations of antidepressant drugs and their targets produce clinical antidepressant effects is unclear. Studies have shown that *BDNF* expression is downregulated in patients with depression and that antidepressant drugs can increase the level of *BDNF* expression (14). When used in combination with *TrkB*, *BDNF* can promote neuronal plasticity and antidepressant response, exert cholesterol sensitivity, and mediate the synaptic effect of cholesterol. In addition, typical and fast-acting antidepressants directly bind to *TrkB*, promoting the synaptic localization of *TrkB* and the activation of *BDNF* (15). Conventional antidepressant drugs, including ketamine, require *BDNF* for the regulation of the antidepressant effects. As a key sensor of antidepressant drugs, *BDNF* may be a biomarker that can be used to monitor depression treatment response (16). At present, it is generally believed that serotonin receptors are closely associated with the development of depression. Recent studies have found that two oligosaccharide esters (3,6’-deadenosucrose *(DISS*) and deadenosyl Ester A (*TFSA*) can be used in combination to effectively enhance the action of the serotonin-*BDNF* pathway to improve the antidepressant effect (17).

*Bipolar disorder*

Bipolar disorder is a chronic disease. Bipolar disorder patients experience significant mood changes that fluctuate between mania and depression. Studies have shown that the concentration of *BDNF* in the serum of patients with bipolar disorder is significantly lower than that of healthy people. After treatment, the concentration of *BDNF* in the peripheral blood of patients with bipolar disorder was found to be significantly higher than that of healthy people, while the concentration of *BDNF* in the serum of patients with bipolar disorder was in remission. There was no significant difference in the concentration of *BDNF*, compared with healthy people (18). Therefore, some studies have suggested that the concentration of *BDNF* in peripheral serum can be used as an index to determine the condition of patients with bipolar disorder and that the change of the concentration of *BDNF* in peripheral serum be used as criteria to determine clinical efficacy during the onset of bipolar disorder (19). *BDNF* levels in bipolar disorder patients were significantly lower than that of the normal control group, which included a healthy control group and a unipolar depression group (20). Valvassori et al. (20) found that the expression of the *BDNF* gene and its protein in the frontal cortex in an animal model of ouabain-induced bipolar disorder was downregulated. A recent study compared *BDNF* levels of newly diagnosed bipolar disorder patients with unaffected first-degree relatives and healthy control groups, and found that *BDNF* levels were downregulated in early-stage bipolar disorder patients but not in unaffected first-degree relatives (21), which points to a new direction for future research.

*Schizophrenia*

Schizophrenia (*SCZ*) is a debilitating mental illness with complex, variable, and high genetic characteristics. Imaging studies have found that the volume of the thalamus, hippocampus formation, prefrontal and orbitofrontal cortex, and amygdala-hippocampus complex of patients with schizophrenia were reduced, accompanied by reduced interleukin 1β (*IL-1β*) and IL-6 expression. The expression of IL-8 and *TNF-α *were upregulated, while the expression of *BDNF* and *TrkB* were significantly downregulated (22). Due to the role played by *BDNF* in neurogenesis and synaptogenesis and its effect on the function of dopaminergic neurons, it can be used as a biomarker of schizophrenia and its treatment (23). Changes in *BDNF* expression and Val66Met (rs6562) polymorphism are involved in the pathogenesis of schizophrenia. A new study clinically evaluated 573 patients with chronic schizophrenia. The results showed that the age of onset and cognitive function of the schizophrenia patients was related to changes in clinical characteristics, with serum *BDNF* levels and *BDNF* Val66met polymorphisms influencing the age of onset of schizophrenia, cognitive function and clinical symptoms (24). Another study found that patients with schizophrenia and *T2DM* (type 2 diabetes) showed elevated *BDNF* levels and improved cognitive function. It was speculated that this change may be related to the pathophysiological processes of chronic *SCZ* patients with *T2DM*. Therefore, these results provide a new basis for future research (25).

*Alzheimer’s disease*

Alzheimer’s disease is a common degenerative disease of the nervous system, which mainly manifests as cognitive dysfunction, for which the cause is still unknown (26). Increasing evidence has shown that *BDNF* is associated with the etiology and pathogenesis of *AD*. Studies have found that *BDNF* exerts a potential protective effect based on neurotoxicity caused by the amyloid β protein (*Aβ*) in AD mouse models, with the serum and brain *BDNF* levels in mice (a mouse model of Tauopathy) found to be downregulated (27). A comparative analysis of* AD* patient blood, cerebrospinal fluid, and post-mortem cranial neurotrophic factor levels with the control group found that peripheral blood *BDNF* levels of *AD* patients were significantly lower than those in the control group, while *BDNF* levels in the hippocampus and neocortex of *AD* patients were significantly downregulated (28). Recent studies have shown that *Aβ* can downregulate *BDNF* levels primarily by downregulating the expression of phosphorylated cyclic adenosine monophosphate (*CAMP*) response element-binding protein (*CREB*). Drugs or gene therapy that can target *CREB*-*BDNF* signaling may be a novel intervention method that can be used to improve the decline in *AD* cognitive function (29). Therefore, these findings provide a basis for further research on the biomarkers and therapeutic targets of *AD*.

*Parkinson’s Disease*

Parkinson’s disease (*PD*) is a common neurodegenerative disease among middle-aged and elderly individuals. The main clinical manifestations of *PD* are reduced movement and resting tremors. A large number of studies have found that *PD *patients have reduced levels of *BDNF* expression in the ventral substantia nigra of the brain. Current studies have found that the protein, tyrosine phosphatase 1B (*PTP1B*) can improve nerve damage by significantly reducing the regulatory effect of interferon γ on inflammatory cytokines and can reverse the 6-hydroxydopamine- (6-OHDA-) induced downregulation of cyclic adenosine phosphate response element-binding protein (*p-CREB*) and *BDNF* in SH-SY5Y cells (30). In PD patients, *BDNF* protects and repairs dopamine neurons, and the *BDNF*Val66Met gene polymorphism is closely associated with *PD* (31, 32). Although there is no evidence that *BDNF* expression is directly associated with *PD*, these studies may provide new targets and strategies for the treatment of *PD*.

*Epilepsy*

Epilepsy is a sudden and dysfunctional brain disease. Studies have found that *BDNF* and its binding receptor, *TrkB*, are upregulated in animal models and that in patients with epilepsy, especially in the temporal and hippocampal regions (33). Seizures also can upregulate protein levels of *BDNF* in the hippocampus and cortex in animal models of epilepsy. Previous studies have shown that *BDNF* and *TrkB* molecules are very promising therapeutic targets for the treatment of epilepsy (34, 35). *BDNF* is closely associated with the pathogenesis of epilepsy, and novel treatment methods that use *BDNF* can be developed. However, it is worth noting that *BDNF* may play a role in protecting neurons from harmful stimuli. Therefore, reductions in *BDNF* levels should be carefully considered when treating epilepsy patients.

*The role of BDNF in cardiovascular and cerebrovascular diseases*

Stroke results from damage to brain tissue caused by the sudden rupture of blood vessels in the brain or when blood cannot flow into the brain due to clogged blood vessels. Stroke is an acute cerebrovascular disease and is of two types, ischemic stroke, and hemorrhagic stroke. The diagnostic significance and clinical value of *BDNF* during the acute phase of stroke are controversial (36). Early studies conducted by Be´jot *et al.* (37) showed that the injection of different doses of microspheres through the carotid artery and into circulation can induce unilateral ischemic stroke in rats, and can be used to simulate the different degrees of stroke severity in stroke patients to explore the significance of circulating *BDNF* levels in stroke patients. Finally, it was found that circulating *BDNF* levels after stroke does not reflect *BDNF* levels during the stroke and that there is a correlation between severe stroke and high plasma *BDNF* levels during the acute phase. A recent study found that a Chinese herbal medicine used for the treatment of stroke, Yizhi (*AOM*), can significantly upregulate the expression of *BDNF* and paromomycin receptor kinase B (*TrkB*) in the hippocampus, thereby enhancing adult hippocampal neurogenesis and improving MCAO deficiency, thereby enhancing spatial learning, memory and cognitive functions in rats (37). The production of endogenous *BDNF* in reactive astrocytes is associated with the onset of stroke in hypertensive rats, while exogenous central *BDNF* causes high blood pressure in hypertensive rats, leading to an increased incidence of stroke. Due to its significant impact on mortality, *BDNF* may be a candidate drug for the treatment of neurovascular diseases in the future (38). A recent study showed that *BDNF* was significantly downregulated in patients with coronary artery calcification compared with normal control individuals. *BDNF* plays an important role in maintaining the stability of vascular endothelial cells and reducing atherosclerosis and can induce the occurrence of coronary atherosclerosis. Therefore, *BDNF* expression can be used for the prediction of certain clinical conditions (39).

*Other applications*

In addition to the applications mentioned above, *BDNF* has also shown good applicability for the treatment of other diseases, such as glaucoma (40), strabismus (41), and irritable bowel syndrome (42). In the future, *BDNF* can be applied in other forms for the treatment of other diseases than used at present.

*Application of a drug delivery system for BDNF*

**Figure 2:**
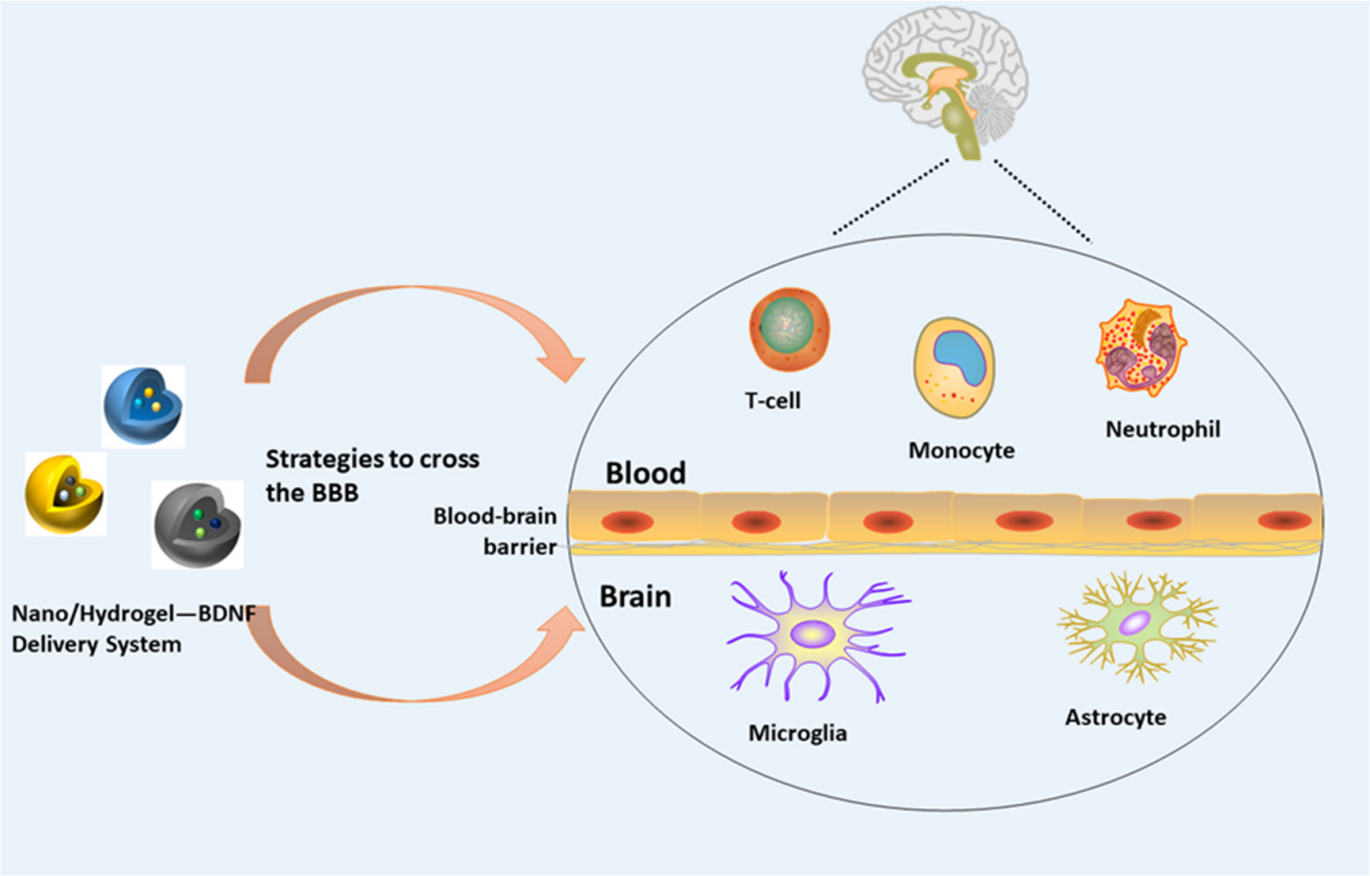
Nano/hydrogel—*BDNF* delivery system and the blood-brain barrier

In recent times, *BDNF* has become a research focus due to its wide range of applications. However, it cannot exert its functions quickly and accurately due to its difficulty in penetrating the blood-brain barrier. However, nano delivery systems and hydrogels can be used to overcome this challenge. Nanoparticles provide a series of unique properties for drug delivery, including high drug loading capacity, combined delivery, controllable and sustained drug release, extended stability and lifespan, and targeted delivery (45) (Figure 2). To further improve the therapeutic index, especially for local applications, nanoparticles have been increasingly combined with hydrogels to form hybrid biomaterial systems for controlled drug delivery, and are now widely used for the treatment of cancer (46, 47), spinal cord injury (48), bone transplantation, and other (49) diseases. In recent years, research on *BDNF* delivery using nanotechnology and hydrogels has made great progress.

*Stem Cell-based BDNF Delivery System*

**Figure 3:**
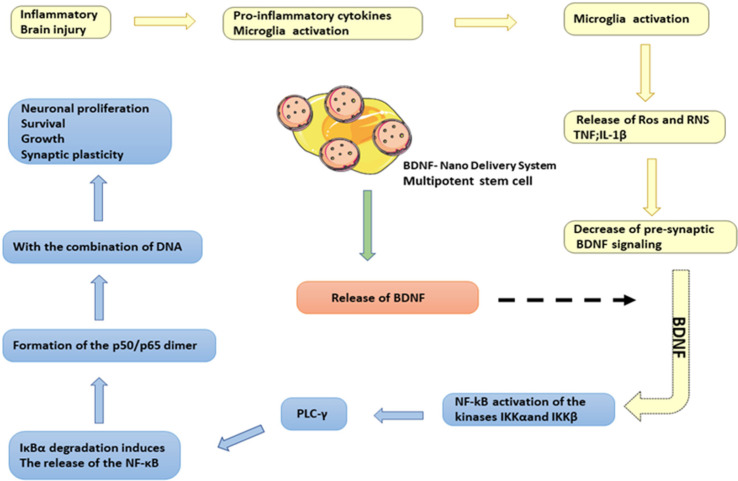
*BDNF*-Stem cell-Nano delivery system. In the case of brain inflammation or brain damage, it will induce the pro-inflammatory activation of NF-kB-dependent microglia and release toxic mediators, such as ROS and RNS, TNF, IL-1β, etc. The *BDNF* signaling pathway in the synaptic space will be reduced. *BDNF*-nano delivery system released *BDNF* and *BDNF* induced the expression of *NF-κB* and stimulates *PLC-γ/PKC* signal by activating IKKα and IKKβ kinases. These kinases phosphorylate the *NF-κB *inhibitory unit IκBα, leading to ubiquitin binding and subsequent degradation of IκBα through the proteasome. *IκBα* degradation induced the release of *NF-κB* and the formation of p50/p65 dimers, which bind to DNA and induced the expression of genes related to neuronal proliferation and differentiation

A variety of biomaterials can be used to encapsulate stem cells as a potential cell transplantation strategy for the treatment of neurological diseases (Figure 3). However, an ideal cell delivery material and method is yet to be developed for the clinical treatment of encephalopathy. Previously, researchers used a chitosan (Chitosan) scaffold based on genipin (*GP*) as a cross-linking agent to fix *BDNF* with human umbilical cord mesenchymal stem cells (*huC-MSCs*) for the delivery of *BDNF* and found that *BDNF* released by *CGB* scaffolds (Chitosan-genipin-*BDNF*) could promote neuronal differentiation of neural stem cells. The *CGB *scaffold shows high biocompatibility with human umbilical cord mesenchymal stem cells, suggesting that the granular *CGB* scaffold covering human umbilical cord mesenchymal stem cells may contribute to the development of better treatment methods for traumatic brain injury (50). Subsequent studies have integrated adult pluripotency induced (*MIAMI*) stem cells isolated from human bone marrow and pharmacologically active microcarriers (PAMs) into an injectable non-toxic silanized hydroxypropyl methylcellulose (*SiHPMC*). In the hydrogel, an injectable non-toxic cell and growth factor delivery device is used for the delivery of *BDNF*. Both *PAMs* and *SiHPMC* hydrogel can promote the survival of transplanted cells and neuronal differentiation and can transport them into the central nervous system to enhance its tissue repair function. As a novel local delivery tool, it provides a promising treatment method that can be used to promote angiogenesis, neuroprotection and axon growth in the nervous system (51).

*BDNF* can enhance the proliferation and differentiation of mesenchymal stem cells into osteoblasts. Polyelectrolyte complex nanoparticles (*PECNP*) have been reported as a suitable drug delivery system. Loy *et al.* (51) studied the effect of *BDNF*-loaded *PECNP* nanoparticles on osteoblasts and found that PECNP is a drug delivery system that is suitable for bone grafts and has no negative effect on bone cell production protein *in-vitro*. A recent study combined dental stem cells (*SCAP*) from the tip of the nipple with pharmacologically active microcarriers *(PAM*) that release *BDNF* and found that *BDNF* improved the motor function of rats through immunomodulation and neuroprotection. Subsequently, the poly-L-lactide-glycolide copolymer was used as a solid/oil/water emulsion to coat *BDNF*, which was then coated with fibronectin to prepare *BDNF*-PAM, and plant *SCAP* on it. *SCAP*-*BDNF*-*PAM* injection into the spinal cord injury site of rats can improve the blood-brain barrier score, reduce the expression of inducible nitric oxide synthase, and increase the expression of βⅢ tubulin, *GAP43*, and 5-HT. These results confirmed the applicability and versatility of PAM as a drug and cell combined drug delivery system in the field of regenerative medicine, and also confirmed the potential therapeutic potential of *BDNF*-*PAMS* for the treatment of spinal cord injury (52). Researchers have also embedded human mesenchymal stem cells (*hMSCs*) into silk fibroin-based hydrogels to produce excess *BDNF*. *BDNF*-*hMSCs* can be transplanted through nasal septal cell transplantation to treat brain injury in rats and was found to significantly reduce the death of hip neurons after injury and promote the recovery of nerve function. Overall, the stem cell transplantation method has laid a good foundation for the clinical applications of *BDNF* for the treatment of brain injury (53).

*Other BDNF delivery systems*

To overcome the challenge of the inability of *BDNF* to pass through the blood-brain barrier, researchers have begun to identify ways to improve the permeability of *BDNF*. At present, studies have used *BDNF* and the BBB modulator (BBBM) peptide, ADTC5, to deliver recombinant brain-derived neurotrophic factors to the brains of healthy mice and experimental autoimmune encephalomyelitis (*EAE*) mice through intravenous injection. The results show that ADTC5 can promote *BDNF* transmission to the brain of healthy SJL/ELITE mice, and trigger *TrkB* receptor phosphorylation in the brain. EAE mice treated with *BDNF*+ADTC5 can inhibit EAE recurrence better than mice treated with *BDNF*, *ADTC5,* or the vehicle alone (54). Surfactant-coated polylactic acid-glycolic acid (*PLGA*) nanoparticles can deliver a variety of molecules across the blood-brain barrier through receptor-mediated endocytosis. Studies have also found that PLGA nanoparticles coated with poloxamer (*PX)* can effectively deliver *BDNF* to the brain, improve neurological and cognitive deficits in mice with traumatic brain injury (*TBI*), and thus exert a neuroprotective effect (55). Lu *et al.* (56) developed a self-assembled peptide nanofiber hydrogel that delivered both vascular endothelial growth factor (*VEGF*) and *BDNF* for peripheral nerve reconstruction. *In-vitro* cell experiments that compared the use of VEGF or *BDNF* mimic peptide epitopes alone, found that functionalized peptide hydrogel scaffolds effectively promoted Schwann cell promyelination and endothelial cell adhesion. The use of functionalized self-assembling peptide nanofiber hydrogels to construct the artificial neurovascular microenvironment in diseased areas has great potential in promoting nerve tissue engineering and other forms of tissue regeneration. Therefore, the study of nano delivery systems that can target and release *BDNF* for a long period is of high importance. Schmidt (56) *et al.* loaded porous silicon nanoparticles (nano single nucleotide polymorphisms) with a diameter of fewer than 100 nanometers with *BDNF* and tested the efficiency of a long-term delivery system for neurotrophins, and the results showed a good level of delivery. Lopes et al. (58) used polymer nanoparticles based on thiolated trimethyl chitosan (*TMCSH*) to mediate the delivery of targeted genes to peripheral neurons through peripheral and minimally invasive intramuscular administration. The addition of non-toxic carboxyl fragment of tetanus neurotoxin (*HC*) onto nanoparticles was used to achieve neuron targeting and to explore the potential application of plasmid DNA encoding brain-derived neurotrophic factors in peripheral nerve injury models. The study found that *TMCSH-HC*/*BDNF* nanoparticle therapy promoted the release and significant expression of *BDNF* in nerve tissues. The results showed that functional recovery after injury was enhanced and key protective factors were improved.

Currently, *BDNF* and biomaterials have been widely used for the treatment of many diseases, in particular spinal cord injuries. Some studies have created novel *BDNF*-loaded cationic nanobubbles (*CNBs*) by combining MAP-2 antibodies (*mAbMAP-2/BDNF/CNBs*) for low-intensity focused ultrasound *(LIFU*) targeted gene therapy. Research has shown that mAbMAP-2/*BDNF*/CNBs can specifically target neurons, and ultrasound targeting transfection of *BDNF* overexpressing neurons can effectively inhibit neuronal apoptosis. In a rat model of acute spinal cord injury, mAbMAP-2/*BDNF*/CNs significantly increased the expression of *BDNF*, reduced tissue damage and neuron loss, while improving the permeability of the blood-brain barrier (57). Ghosh (58) and others have developed a novel hydrogel-based system that can be loaded with polysaccharide-*BDNF* particles self-assembled through electrostatic interactions, which can be safely delivered to spinal cord injury sites, while the dosage and administration time can be controlled. A study suggested that local *BDNF* hydrogel delivery is a very effective and safe strategy that can be used to restore diaphragm function after spinal cord injury. The same research study produced a gelatin-genipin hydrogel system impregnated with ionic liquid that can be injected into the spinal cord injury site of rats. It was found that the survival rate of neurons increased significantly along with the volume of activated microglia and damage was reduced, thereby reducing the level of secondary damage (59). Some researchers have used poly (lactic-glycolic acid copolymer) (*PLGA*) as a carrier to encapsulate *BDNF* and create an injectable gel, which can continuously deliver *BDNF* to the spinal cord injury site for repair (60).

At present, researchers have successfully immobilized insulin-like growth factor-1 (*IGF-1*) and (*BDNF*) on biodegradable graphene oxide (*GO*) composite PLGA (*PLGA/GO*) to create spinning nanofibers and study their effect on nerve regeneration. The results show that IGF-1 and *BDNF* loaded on *PLGA/GO *nanofibers can not only protect NSC from oxidative stress induced by H_2_O_2_ but can also promote NSC proliferation and neuronal differentiation. PLGA/GO is an effective carrier for the delivery of IGF-1 and *BDNF*, and that IGF-1 and *BDNF* immobilized on *PLGA/GO* nanofibers can function as neural implants, showing a high potential for the treatment of spinal cord injury (61). Another study evaluated the release potential of *BDNF* from *IKVAV* functionalized peptide amphiphilic (PA) self-assembled nanofibers and hydrogels, and used *IKVAV-PA *hydrogels loaded with *BDNF* to perform treatment on mice with severe spinal cord injury. The treatment results showed that *IKVAV-PA* is arranged into a nanofiber structure and can continuously release *BDNF* while retaining the biological activity of *BDNF*. Injection of *IKVAV-PA* hydrogel containing *BDNF* can increase axon retention and decrease levels of astrocyte proliferation after spinal cord injury without any inflammatory response. These results suggest that biofunctional peptide-based hydrogel can be used as an injectable scaffold for *BDNF* delivery to promote regeneration following spinal cord injury (62). Researchers have also developed a self-assembled peptide nanofiber hydrogel based on the self-assembled backbone *Ac- (RADA)4-NH2 (RAD)*, which uses laminin-derived motifs IKVAV (*IKV*) and *BDNF*. The mimetic peptide epitope RGIDKRHWNSQ (RGI) performs the dual functions of peripheral nerve regeneration and enhanced remyelination, as well as motor function recovery (63).

The latest study conducted on *BDNF* application used polymer-based polyethylene glycol-polyaspartic acid (*DET*) nano micelles and its derivatives to administer *BDNF*-encoded mRNA into the brain, which significantly increased transient whole brain expression of *BDNF*. The results showed an increase in the survival rate of hippocampal neurons after ischemia (*TGI*) and a rapid increase in *BDNF* content in the hippocampus after administration. The BNDF-mRNA introduced through the use of multiple types of nano glue can effectively treat ischemic neuronal death (64). Due to the limitation of the BBB ​​for drug delivery to the central nervous system (*CNS*) and the safety of intracerebral administration, the intranasal route of administration has recently received extensive attention. Some researchers have dissolved *BDNF* in PBS and administered it as a non-invasive nasal injection to AD11 mice, and have found that it can effectively improve the memory performance of the experimental mice. This increases the potential therapeutic use of *BDNF* for the treatment of neurological diseases and enhances its potential administration through non-invasive intranasal pathways as a brain delivery strategy for *BDNF* and other neurotrophic factors (65). This study used minimally invasive nose bank (Mind) technology to deliver the entire therapeutic dose directly to the sub-olfactory submucosal space through nasal administration, which overcomes dose variability and efficiency-related challenges faced during traditional local transnasal administration. The study found that implanting a reservoir containing AntagoNAT (*AT*), which can downregulate the expression of which can down regulate the expression of *BDNF*, significantly increased the central nervous system distribution of AT and continue to upregulate *BDNF*. In the future, this technology can be used at treatment centers as a potential treatment method for neurological diseases (66).

Previously, researchers had used hyaluronic acid hydrogel loaded with *BDNF* for nerve repair in patients at the chronic phase after stroke, and found that *BDNF* can be released locally from the infarct cavity. In two mouse stroke models, the duration of the spread of *BDNF* delivered through a hydrogel from the stroke cavity to the surrounding tissues of the infarction was longer than that of a simple injection of *BDNF*, and the administration of the *BDNF* hydrogel was able to promote the recovery of motor function (67). Jimbo *et al.* (70) used high molecular weight hyaluronic acid (*HMW-HA*) and BDN to study the regenerative effect of *BDNF* on bifurcation defects in non-human primate models. The study found that the periodontal tissue regeneration level and the formation of acellular cementum were significantly higher in the *BDNF* and *HMW-HA *combined treatment group than that in the *BDNF* alone group.

Many of the drug delivery mechanisms that are currently being studied, for example, using the endothelial transport barrier to deliver drugs into *CNS*. Surfactant-coated nanoparticles can deliver a variety of molecules through the blood-brain barrier by receptor-mediated exocytosis. Nano / hydrogel-*BDNF* delivery system can cross the blood-brain barrier more safely, accurately and quickly.

**Table 1:** The performance of brain-derived neurotrophic factors in diseases

**Type of disease**	***BDNF***	**references**
Major depressive disorder	The levels of *BDNF* in the serum and brain of patients with depression are downregulated; *BDNF* can be used in combination with *TrkB*; traditional antidepressants regulate the level of *BDNF*	(14-16)
Bipolar disorder	The level of *BDNF* in the serum is downregulated during the attack period; the level of *BDNF* expression at gene and protein levels is also downregulated in the frontal cortex	(18, 43)
Schizophrenia	The expression of the *BDNF* gene is downregulated in the hippocampus, thalamus, and frontal cortex of patients; *BDNF*Val66Met gene polymorphism is involved in the pathogenesis	(22, 24)
Alzheimer's disease	The level of *BDNF* in the serum is downregulated; the level of *BDNF* mRNA in the hippocampus is also downregulated	(27, 28)
Parkinson	The *BDNF* content of the ventral substantia nigra of the brain is downregulated in patients; *BDNF*Val66Met gene polymorphism is closely associated with *PD*	(30-32)
Epilepsy	Seizures increase protein levels of *BDNF* in the hippocampus and cortex	(34, 35)
Cardiovascular	The diagnostic significance and clinical value of *BDNF* during the acute phase of stroke are still controversial; the severe stroke is associated with high plasma *BDNF* levels during the acute phase; exogenous central *BDNF* expression increases blood pressure in hypertensive rats; *BDNF* levels are significantly downregulated in patients with coronary artery calcification	(37-39, 44)

**Table 2:** Delivery systems for *BDNF* using hydrogel/nanomaterials

**Disease application**	**Hydrogel/Nanomaterial**	***BDNF*** ** system**	**References**
Traumatic brain injury and recovery after injury	Chitosan-Genipin; Silk Fibroin (*SF*); Polylactic Acid-Glycolic Acid (*PLGA*); Thiolated Trimethyl Chitosan (*TMCSH*)	Chitosan-genipin-*BDNF*; Silk fibroin-encapsulated human mesenchymal stem cells (*hMSCs*) release *BDNF*; PLGA-*BDNF*; TMCSH-*BDNF*;	(50, 53, 55 and 68)
Promote angiogenesis, neuroprotection and axon growth	Silanized hydroxypropyl methylcellulose (*Si-HPMC*) and pharmacologically active carrier (*PAM*); self-assembled peptide nanofiber hydrogel- (*Beaver Nano RAD*);	*PAM-Si-HPMC*-*BDNF*； *RAD*-*BDNF*	(51, 69)
Rat motor function	Polyelectrolyte composite nanoparticles (*PECNP*)	*PECN-* *BDNF*	(70)
Spinal cord injury	Precipitation of poly-L-lactide-glycolide copolymer, and then combined with pharmacologically active microcarriers (*PAM*); cationic nanobubbles (*CNBs*); polysaccharides; polylactic acid-glycolic acid copolymers (*PLGA*); graphene oxide ( *GO*) Composite *PLGA* (*PLGA/GO*) electrospun nano; *IKVAV *functionalized amphiphilic PA self-assembled nano; Ac- (*RADA*)4-*NH2* (*RAD*) self-assembled peptide nanofiber hydrogel	*BDNF*-*PAMS*; Combined *MAP-2* antibody mAb *MAP-2*-*BDNF*-CNBs; Polysaccharide-*BDNF*; *PLGA-BDNF; PLGA/GO-BDNF; IKVAV-BDNF; c- (RADA) 4-NH2 (RAD) -BDNF;*	(52, 57, 58 and 60-63)
Ischemic neurodeath	Polyethylene glycol-polyaspartic acid (*DET*) nano micelles and their derivatives	*PEG-PAsp (DET）*-*BDNF*	(64)
Stroke	Hyaluronic acid	Hyaluronic acid-*BDNF*	(67)
Periodontal regeneration	Hyaluronic acid *HMW-HA*	*HMW-HA*-*BDNF*	(71)

## Conclusion

Since *BDNF* plays a key role in the development and maturation of neurons, it has become the main therapeutic drug target for many neurological diseases. A large number of studies have shown that *BDNF* has shown potential therapeutic value in many neurodegenerative disease models and acute central nervous system damage. However, due to transmission challenges associated with this molecule, its clinical application is not perfect. In addition, the positive effects of psychotropic drugs can activate *BDNF*-mediated signal transmission. Although the *BDNF* gene is associated with many diseases, in addition to functional genetic variation, other molecular mechanisms may also affect the regulation of the expression of the *BDNF* gene, resulting in *BDNF* signal transduction disorders and related pathological changes. *BDNF* plays a key role in synaptic plasticity, partly due to changes in local protein synthesis. The activation of *BDNF* by *TrkB* can trigger multiple parallel signaling pathways, such as the *RAS/ERK*, phosphatidylinositol 3-kinase, and phospholipase C-γ pathways. Although *BDNF* can regulate certain signaling mechanisms of translation activity by regulating the initiation and expansion stages, changes in the proteome caused by *BDNF* have not been verified.

To overcome the challenge of poor blood-brain barrier permeability, bone marrow mesenchymal stem cells can be used as a delivery platform for *BDNF* to provide broad prospects for clinical neurotrophic factor repair. As in-depth research continues to be conducted on nano-bio materials, an increasing number of researchers have aimed to identify hydrogel materials and nanotechnology that can be used for the targeted delivery of *BDNF*, to achieve rapid, accurate, safe and continuous delivery of *BDNF*. At present, a variety of nanosystems, such as self-assembled nanometers, biodegradable materials, and injectable gels, have been applied for the delivery of *BDNF*. However, current disease models can usually only simulate certain symptoms, and cannot accurately reproduce complex human diseases. Therefore, intrathecal injection of *BDNF* into the brain or spinal cord of patients as an intervention is still accompanied by many challenges. The studies and investigations detailed above provide novel methods and a foundation for the application of *BDNF* for the treatment of various diseases in the future.

## Conflicts of Interest

The authors declare that there is no conflict of interest.
